# Protective Effects of Aqueous Extract of *Solanum nigrum* Linn. Leaves in Rat Models of Oral Mucositis

**DOI:** 10.1155/2014/345939

**Published:** 2014-11-20

**Authors:** Alkesh Patel, Subhankar Biswas, Muhammed Haneefa Shoja, Grandhi Venkata Ramalingayya, K. Nandakumar

**Affiliations:** Department of Pharmacology, Manipal College of Pharmaceutical Sciences, Manipal University, Manipal, Karnataka 576104, India

## Abstract

Oral mucositis is one of the most debilitating side effects in patient undergoing chemotherapy or chemoradiotherapy. Leaves of the plant *Solanum nigrum* are used in folklore medicine to treat oral ulcers in India. However, no pharmacological investigation has been carried out till date. Aqueous extract of *Solanum nigrum* leaves (AESN) was prepared and subjected to various phytochemical screening. HPLC analysis of the ethyl acetate fraction was carried out. The aqueous extract (100 and 200 mg/kg) was further evaluated for its protective effect on two rat models: (a) busulfan plus infrared radiation (chemoradiotherapy) induced oral mucositis and (b) methotrexate (chemotherapy) induced oral mucositis. Various parameters including body weight change, food intake, and mortality were measured. AESN showed protective effect in both models of oral mucositis; however, the higher dose was more effective in chemotherapy induced oral mucositis. A reduction in oral mucositis score (*P* < 0.05) was observed in the treatment groups. Significant (*P* < 0.05) improvement in food intake was also observed in AESN treated groups. Aqueous extract of *Solanum nigrum* leaves has protective effect on chemotherapy and chemoradiotherapy induced oral mucositis in rats.

## 1. Introduction

Oral mucositis is a common complication of chemotherapy and radiotherapy to the head and neck region. It is often a dose limiting toxicity prohibiting the patient from receiving optimal therapy. Almost every patient with oral cancer treated with chemoradiotherapy develops deep and painful wounds characteristic of this condition [1]. The chance of oral mucositis occurrence ranges from 30 to 40% in the general cancer patient population [2]. Conversely, there is a chance of developing 76% of oral mucositis in patients undergoing high-dose chemotherapy conditioning before hematopoietic stem cell transplant [3]. Mucositis is the second most frequent dose limiting toxicity in patients receiving chemotherapy [4]. For instance, 40% of patients treated with fluorouracil based regimens and >60% of patients receiving bone marrow transplant conditioning therapies for hematological or solid tumors develop this toxicity [5, 6].

For over a decade, complementary and alternative medicine (CAM) has been in use by cancer patients in western countries (USA, Canada, Germany, etc.) with prevalence as high as 80% [7, 8]. Among CAM, traditional Chinese medicine (TCM) is of higher importance, as it is based on a holistic, natural approach and well established theoretical framework. Although CAM has potential in treating various disorders, lack of scientific data has kept it behind conventional therapies. However, recent studies have investigated CAM for their potential in alleviating the toxicity associated with chemotherapy induced mucositis [9]. Here the plant,* Solanum nigrum,* commonly known as black nightshade, is frequently used as a valuable ingredient for clinical TCM cancer therapy [10]. Chinese people have used the leaves of this plant to alleviate inflammation and edema through its antipyretic and diuretic effect [11]. Hepatoprotective activity of this plant is well established [12]. Studies have been conducted providing evidence for the antitumor potential of this plant, including liver, stomach, lung, bladder, breast, and colon cancer [10]. Leaves of this plant have been used as traditional folk medicine by the inhabitants of southern India to treat mouth ulcers. To our knowledge, no study has been conducted to determine the protective effect of this plant for oral mucositis. Therefore, the purpose of this study was aimed at estimating the protective effect of* Solanum nigrum* leaves against chemotherapy and chemoradiotherapy induced oral mucositis in rats.

## 2. Materials and Method

### 2.1. Extraction and Phytochemical Analysis of Leaves of* Solanum nigrum*

#### 2.1.1. Plant Material

Fresh plant was obtained from Mahaveer Trading Company, Madurai, Tamil Nadu, and sample of the plant leaf was authenticated by Dr. K Gopalakrishna Bhat, Professor of Botany, Poorna Prajna College, Udupi, Karnataka, India.

#### 2.1.2. Preparation of Aqueous Extract of* Solanum nigrum*

Dried leaf powder of* Solanum nigrum* was macerated with chloroform water. The mixture was stirred frequently at an interval of 3 h. After 3 days, solvent was replaced with fresh solvent and maceration was performed as mentioned above for 2 times. The filtrate was filtered through muslin cloth followed by Whatman no. 1 filter paper to avoid fine powder. The extract was concentrated using rotary evaporator. Finally aqueous extract of* Solanum nigrum* (AESN) obtained was stored in vacuum desiccators after freeze drying. The percentage yield was found to be 10%.

#### 2.1.3. Preliminary Phytochemical Analysis

Various phytochemical analyses were carried out on AESN to determine the presence of phytoconstituents. Screening was performed for anthraquinones, resins, flavonoids, saponins, tannins, and alkaloids using the standard procedure [13]. The color intensity or the precipitate formed was used as analytical response for these tests.

#### 2.1.4. High-Performance Liquid Chromatography (HPLC) Characterization

HPLC analysis was carried out according to a previously described method [14] with few modifications. In brief, aqueous extract was fractionated with ethyl acetate and was dissolved in methanol (1 mg/mL) before subjecting to analysis. The HPLC system (Shimadzu, Japan) consisted of dual pump LC-20AD binary system, PDA detector SPD-M20A; Merck C_18_ reversed-phase column (I.D. 4.6 mm × 250 mm, 5 *μ*m). Separation was carried out using a two-pump linear gradient program for pump A (40% acetonitrile) and pump B (5% acetonitrile). Elution was initiated with a gradient of 12% A changing to 40% in 20 min, 100% in 45 min and finally to 12% in 55 min followed by washing for 35 min. Injection volume was 20 *μ*L and flow rate was maintained at 1 mL/min throughout the process.

### 2.2. *In Vivo* Studies

#### 2.2.1. Animals

Animal care and handling was carried out in accordance with the Committee for the Purpose of Control and Supervision of Experiments on Animals guidelines after research project approval by Institutional Animal Ethical Committee, clearance certificate no. IAEC/KMC/70/2011-2012. Female Wistar rats inbred at Central Animal Research Facility, Manipal University, Manipal, were used for the study. They were maintained under controlled condition of temperature (23 ± 2°C), humidity (50 ± 5%), and light (14 and 10 h of light and dark, resp.). The animals were provided with food and water* ad libitum. *Rats were housed in sterile polypropylene cages containing sterile paddy.

#### 2.2.2. Toxicological Study

Toxicity study was carried out to determine the safe dose as per Organization for Economic Co-operation and Development (OECD) 425 guideline. Limit test was performed at 2000 mg/kg, p.o. using 5 animals. Extract was administered to 6 h fasted mice. After administration the animals were observed continuously for 1 h, followed by which animals were observed at 4th and 24th h. After administration, Irwin's test was conducted in which animals were observed for gross behavioral changes like awareness, alertness, irritability, passivity, grooming, and restlessness. Neurological profile like spontaneous activity, reactivity and tremor, touch and pain response was also evaluated. Autonomic profile like writhing, defecation, and urination was evaluated.

### 2.3. *In Vivo* Antioral Mucositis Screening

#### 2.3.1. Preparation of Test Compound and Standard Drug

*Test Compound*. The aqueous extract of* Solanum nigrum* (AESN) was dissolved in sufficient quantity of water and was administered orally to rats at a dose of 10 mL/kg.

*Standard Drug*. Sufficient quantity of L-glutamine powder was mixed with 3% of tween 80, triturated, and administered orally.

#### 2.3.2. Procedure for Induction of Oral Mucositis

*(1) Chemotherapy and Radiation Induced Oral Mucositis*. In this model of mucositis, both chemotherapy and infrared radiation were used for induction of oral mucositis according to the method described previously [15]. The chemotherapeutic drug used was busulfan (purchased from Sigma-Aldrich) at a dose of 6 mg/kg for 4 days by oral route. The means of radiation was infrared at an intensity of 40 mV/cm^2^ for 5 seconds using tail flick apparatus (Ugo Basil, model 37360, Italy) on 1st, 4th, and 10th day on the dorsal surface of rat tongue during the experiment period. Each rat was anaesthetized before radiation exposure which reduced the sensitivity of pain and provided adequate relaxation of tongue for exposure to infrared source.

*(2) Methotrexate Induced Oral Mucositis.* Induction of mucositis was achieved by the procedure described previously [16, 17] with few modifications. In brief, methotrexate (purchased from United Biotech) 2.5 mg/kg was dissolved in phosphate-buffer saline (PBS) and injected subcutaneously each day for three consecutive days. Induction of mucositis along with formation of proinflammatory cytokines was validated previously by this model [18].

#### 2.3.3. Selection of Doses

Two doses of AESN (100 and 200 mg/kg) were used based upon the result from acute toxicity study and were administered once daily by oral route.

#### 2.3.4. Experimental Groups

*Group 1 (normal control).* Animals (*n* = 6) received tween 80 (3% v/v) in water p.o.

*Group 2 (mucositis control).* Mucositis induction by busulfan and radiation (*n* = 6) or with methotrexate (*n* = 6) received only vehicle {tween 80 (3% v/v)} in water p.o.

*Group 3 (standard).* Animals pretreated for 3 days with L-glutamine (1 g/kg p.o.) followed by mucositis induction with busulfan and radiation (*n* = 6) or with methotrexate (*n* = 6) along with L-glutamine administration for 15 days. 

*Group 4 (AESN).* Animals pretreated for 3 days with AESN (100 mg/kg, p.o.) followed by induction of mucositis with busulfan and radiation (*n* = 6) or with methotrexate (*n* = 6) along with AESN (100 mg/kg, p.o.) treatment for next 15 days. 

*Group 5 (AESN).* Animals pretreated for 3 days with AESN (200 mg/kg, p.o.) followed by induction of mucositis with busulfan and radiation (*n* = 6) or with methotrexate (*n* = 6) along with AESN (200 mg/kg, p.o.) treatment for next 15 days.

#### 2.3.5. Parameters Assessed during Study Period

*(1) Body Weight and Food Intake*. Body weight and food intake were observed regularly in each group during 15 days of study. For determination of food intake, animals were housed individually and 15 g of food pellet was kept in each cage and after 24 h left-over food was measured. 

*(2) Oral Mucositis Score.* Different scoring system was used for scoring oral mucositis on dorsal surface of rat tongue mucosa. A previously described scoring system [19] was used for busulfan plus infrared radiation induced oral mucositis scoring (Table 1). The scoring system used for methotrexate induced mucositis is shown in Table 2. 

*(3) Mortality Rate*. Mortality rate among the groups was determined during 15 days of study period and % mortality for each group was calculated accordingly.

*(4) Hematological Parameter*. Blood count was measured on 9th day of study period in busulfan and infrared radiation induced oral mucositis model, while in methotrexate induced oral mucositis model blood count was determined on 7th day and 12th day of study period by veterinary blood cell counter (model PCE-210 VET from ERMA INC., Tokyo).

*(5) Histopathology.* Rats of standard and normal group were sacrificed on 15th day from the initiation of treatment. Tongue specimens were collected and stored in 10% neutralized buffered formalin and processed for histopathological findings. Tongue specimen was also collected from mucositis control group if they died before 15th day and was processed similarly.

#### 2.3.6. Statistical Analysis

All values were expressed as mean ± SEM (standard error of mean) for 6 animals in each group. Data were analyzed using one-way ANOVA followed by Tukey's multiple comparison tests using Prism 5.03 (Graph Pad Software Inc., La Jolla, CA, USA). Score for oral mucositis was analyzed using Kruskal-Wallis test followed by Dunn's multiple comparison tests.

## 3. Results

### 3.1. Phytochemical Analysis

Phytochemical screening of AESN revealed the presence of alkaloids, flavonoids, steroids, saponins, tannins, phenols, and anthraquinones.

### 3.2. HPLC Characterization

**Figure 1:**
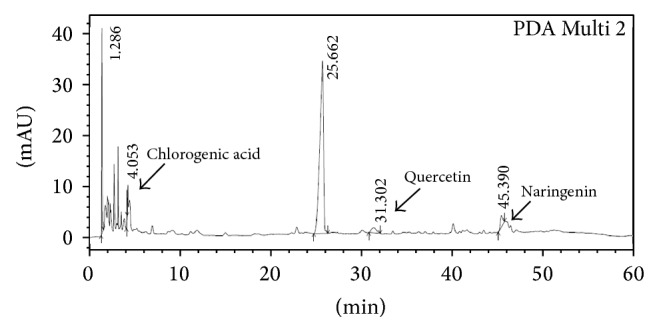
HPLC analysis of ethyl acetate fraction of* Solanum nigrum* Linn. The HPLC used a 4.6 mm × 250 mm, 5 *µ*m Merck C_18_ reversed-phase column. The chromatogram was recorded at 295 nm (PDA Multi 2).

Ethyl acetate fraction was examined for the presence of various flavonoids using HPLC. Individual constituents were identified by comparing their peaks, UV spectra, and retention times (RT), with corresponding reference standards. Flavonoids present in the extract were chlorogenic acid (RT 4.053), quercetin (RT 31.302), and naringenin (RT 45.39) along with some unidentified peaks (Figure 1).

### 3.3. Toxicological Study

The limit test for acute toxicity study showed that AESN was tolerated at a dose of 2000 mg/kg p.o. without any change in normal behavior. No mortality was observed during 72 h and thereafter up to 14 days of observation.

### 3.4. Busulfan and Radiation Induced Oral Mucositis

#### 3.4.1. Body Weight

**Figure 2:**
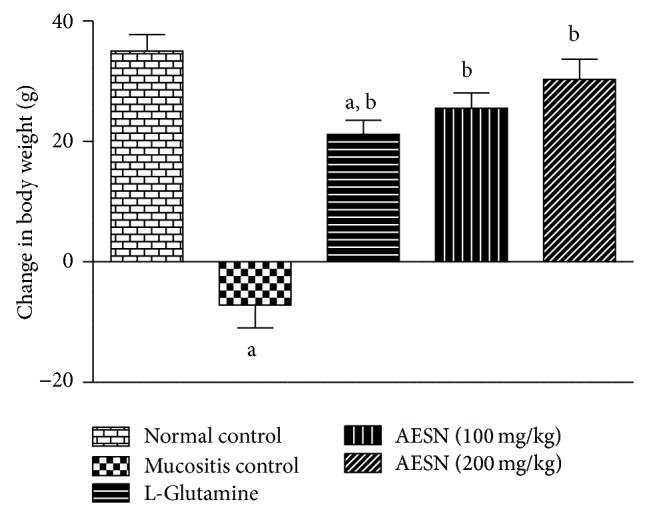
Body weight changes in busulfan and radiation induced oral mucositis. Body weight change in each group was recorded from day 1 to day 15. All values are expressed as mean ± SEM of 6 animals. ^a^ *P* < 0.05 versus normal control, ^b^ *P* < 0.05 versus mucositis control.

Normal control showed increase in body weight of about 16 to 20% over a period of 15 days. Busulfan plus infrared radiation considerably reduced the body weight nearly 1 to 10% in mucositis control on 15th day of study. Treatment with standard and two doses of test compound showed progressive increase in body weight during 15 days of study period (Figure 2).

#### 3.4.2. Food Intake

**Figure 3:**
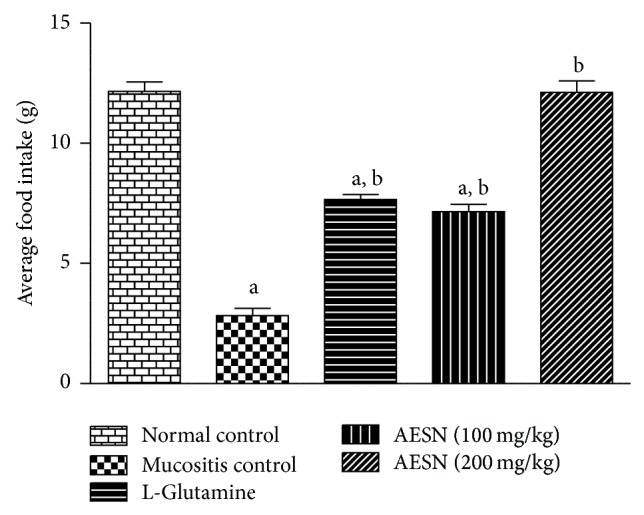
Average food intake in busulfan and radiation induced oral mucositis. 15 g of food was provided for each animal of five groups till the end of study period. All values are expressed as mean ± SEM of 6 animals. ^a^ *P* < 0.05 versus normal control, ^b^ *P* < 0.05 versus mucositis control.

The average food intake in normal control group was found to be 12.16 ± 0.40 g during study period. Mucositis control showed progressive decline in food intake after busulfan and infrared radiation treatment. Treatment with standard and two doses of test compound showed significant (*P* < 0.05) improvement in food intake compared to mucositis control (Figure 3).

#### 3.4.3. Oral Mucositis Score (OMS)

**Figure 4:**
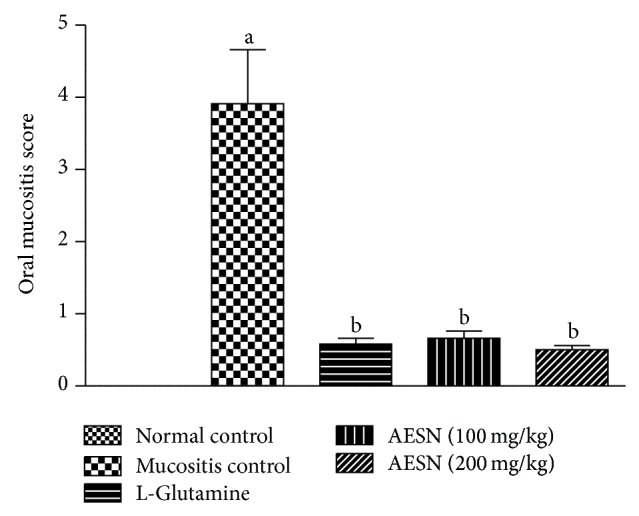
Oral mucositis score in busulfan and radiation induced oral mucositis. Oral mucositis score was measured on a scale of 0 to 5. Scores were analyzed using Kruskal-Wallis test followed by Dunn's multiple comparison tests. All values are expressed as mean ± SEM of 6 animals. ^a^ *P* < 0.05 versus normal control, ^b^ *P* < 0.05 versus mucositis control.

The score for normal group was zero as no mucositis was observed. However, mucositis control showed presence of clear ulceration in 4 out of 6 rats and was recorded as maximum score of 5.0. Treatment with L-glutamine and test compound showed significant (*P* < 0.05) reduction in OMS as compared to mucositis control (Figure 4).

#### 3.4.4. Mortality Rate

**Figure 5:**
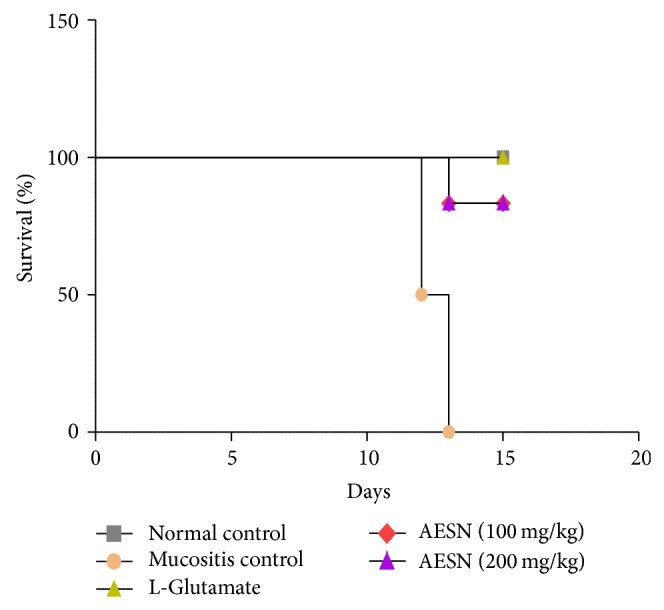
Mortality rate in busulfan and radiation induced oral mucositis. 0% mortality was observed in normal control and L-glutamate groups. However, 100% mortality was seen in mucositis control group on 13th day. Mortality in both the treatment groups was found to be 17% at the end of study period.

Mortality rate was determined during the study period of 15 days. In normal control mortality rate was 0% whereas, in mucositis control, 50% mortality was observed on 12th day and on 13th day of study period the mortality rate was 100%. In contrast, rats in standard group were all alive leading to 0% mortality till the end of study period and only 17% mortality was observed in each of the two doses of test compound throughout the study period (Figure 5).

#### 3.4.5. Hematological Parameters

Blood components in normal control group were within the normal range. However, a significant (*P* < 0.05) decrease in leukocyte (WBC) and platelets count was observed in mucositis control compared to normal. Treatment with standard and test compound showed improvement in leukocyte count (as there was an increase in WBC counts) compared to mucositis control. However, this improvement was insignificant. No significant (*P* < 0.05) improvement was observed in platelet count in treatment groups as compared to mucositis control (Table 3).

#### 3.4.6. Histological Findings

**Figure 6:**
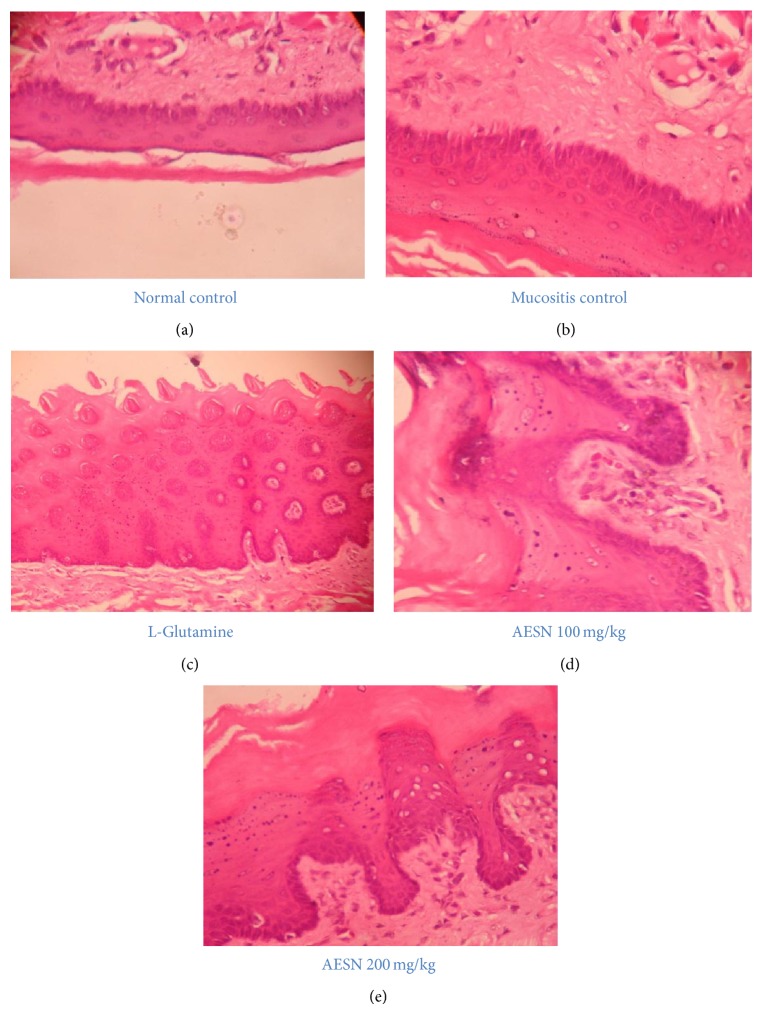
Histology of tongue section of different groups after busulfan/infrared irradiation. (a) Normal control, (b) mucositis control, (c) L-glutamine, (d) AESN 100 mg/kg, and (e) AESN 200 mg/kg. Magnification 40x.

Normal control showed intact epithelium, no lymphocytic infiltration, and normal number of blood vessels (Figure 6(a)). However, in mucositis control, the thickness of epithelium layer was altered and lymphocytic infiltration was observed along with reduction in average number of blood vessels indicating presence of oral mucositis (Figure 6(b)). Treatment with standard and test compounds protected the epithelial layer and showed normal number of blood vessels and absence of lymphocyte infiltration (Figures 6(c), 6(d), and 6(e) resp.).

### 3.5. Methotrexate Induced Oral Mucositis

#### 3.5.1. Body Weight

**Figure 7:**
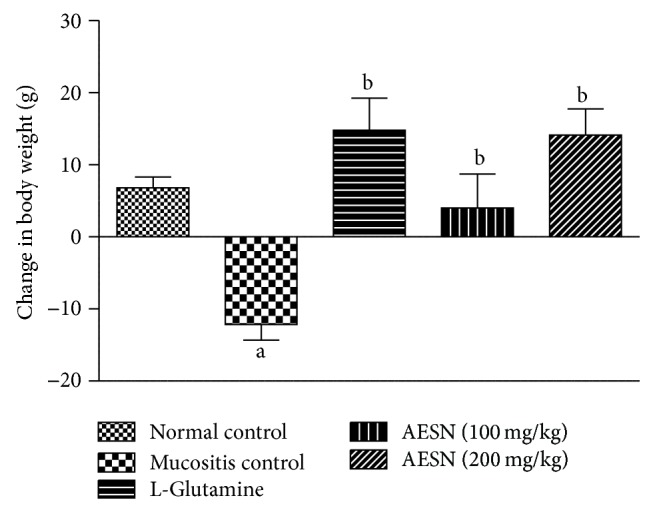
Body weight change in methotrexate induced oral mucositis. Body weight change in each group was recorded from day 1 to day 15. All values are expressed as mean ± SEM of 6 animals. ^a^ *P* < 0.05 versus normal control, ^b^ *P* < 0.05 versus mucositis control.

Increase in body weight of about 3 to 8% over a period of 15 days was observed in normal control group, whereas methotrexate treatment considerably reduced the body weight nearly 3 to 9% in mucositis control during the study period. Treatment with standard and test compound showed progressive increase in body weight during the study period after methotrexate treatment (Figure 7).

#### 3.5.2. Food Intake

**Figure 8:**
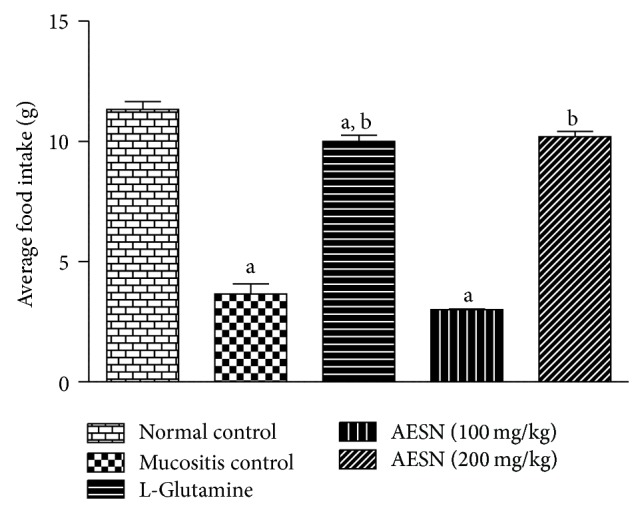
Average food intake in methotrexate induced oral mucositis. 15 g of food was provided for each animal of five groups till the end of study period. All values are expressed as mean ± SEM of 6 animals. ^a^ *P* < 0.05 versus normal control, ^b^ *P* < 0.05 versus mucositis control.

The average food intake of normal control group was found to be 11.3 ± 0.33 g during the study period. Mucositis control group showed progressive decline in food intake after methotrexate administration. Treatment with L-glutamine and AESN (200 mg/kg) showed significant (*P* < 0.05) increase in food intake compared to mucositis control whereas treatment with AESN (100 mg/kg) did not show improvement in food intake compared to mucositis control (Figure 8).

#### 3.5.3. Oral Mucositis Score (OMS)

**Figure 9:**
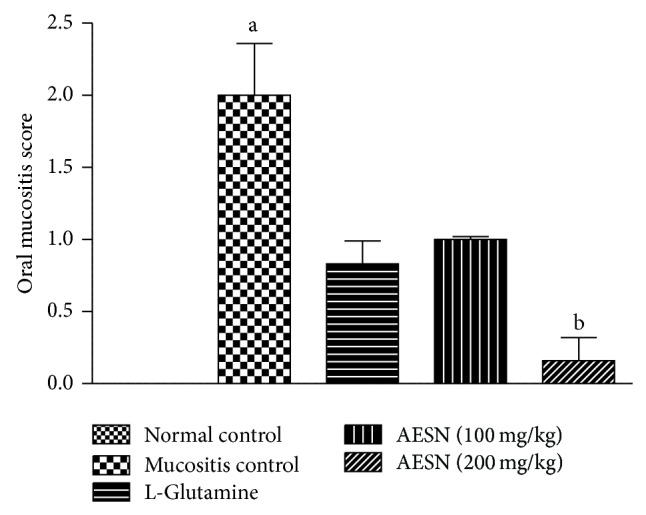
Methotrexate induced oral mucositis score. Oral mucositis score was measured on a scale of 0 to 3. Scores were analyzed using Kruskal-Wallis test followed by Dunn's multiple comparison tests. All values are expressed as mean ± SEM of 6 animals. ^a^ *P* < 0.05 versus normal control, ^b^ *P* < 0.05 versus mucositis control.

Normal control exhibited a score of zero which indicates absence of oral mucositis. However, presence of severe redness of tongue mucosa was observed in mucositis control group indicated by the highest score of 3.0. Treatment with standard and test (AESN 100 mg/kg) did not show significant (*P* < 0.05) difference of OMS compared to mucositis control whereas AESN (200 mg/kg) showed significant (*P* < 0.05) reduction in OMS compared with mucositis control (Figure 9).

#### 3.5.4. Mortality Rate

**Figure 10:**
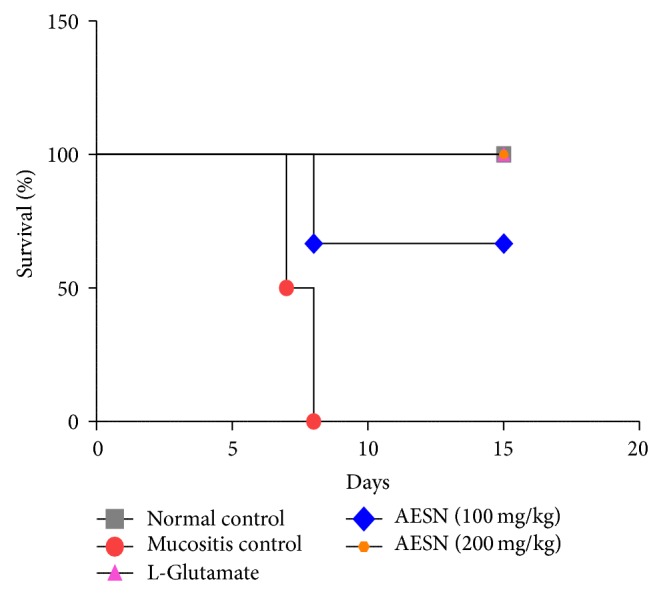
Mortality rate in methotrexate induced oral mucositis. 0% mortality was observed in normal control and L-glutamate groups, whereas in mucositis control group 100% mortality was observed on 8th day. In AESN (100 mg/kg and 200 mg/kg) the mortality rate was found to be 33% and 0%, respectively, at the end of study period.

In normal control, all animals survived the entire study period and the mortality was 0%. In mucositis control, 50% mortality was observed on 7th day and on 8th day the mortality rate was 100%. No death was observed in standard group during the entire study period leading to 0% mortality. In AESN (100 mg/kg) the mortality rate was 33% on 8th day and it was constant till the end of study period. In AESN (200 mg/kg) none of the animals died during the study period leading to 0% mortality (Figure 10).

#### 3.5.5. Hematological Parameters

All blood components were within the normal range in normal control group. On day 7th in mucositis control, WBC and platelets count showed significant (*P* < 0.05) decrease compared to normal control. Treatment with standard and test compound showed progressive increase in WBC count compared to mucositis control; however, the improvement was insignificant. RBC count did not decrease significantly in any of the groups on 7th day (Table 4). In contrast, during 12th day of study period WBC count and platelets showed improvement in the treatment groups compared to mucositis control. However, a slight decrease in RBC count was observed among the treatment groups compared to normal (Table 5).

## 4. Discussion

Chemotherapy and radiation therapy remain the most widely used intervention for the treatments of cancer. Although employed to improve patient's quality of life, severe adverse effects due to these therapies limit their uses. Annually there are approximately 400,000 cases of treatment induced damage to the oral cavity [20]. At present, palifermin (recombinant keratinocyte growth factor-1) has shown to reduce the complications associated with cancer treatment related oral mucositis [21], although several others are in the drug discovery pipeline [22, 23]. Natural products are used in traditional system of medicine to relieve oral ulcer; however, it is not well documented.

In this study, we demonstrated that aqueous extract of* Solanum nigrum* (AESN), a traditional Chinese medicine, has preventive effect against chemotherapy and chemoradiotherapy induced oral mucositis. The preliminary phytochemical screening of AESN revealed the presence of tannins, alkaloids, saponins, flavonoids, phenols, and anthraquinones. Various plant based products have been identified having potential for treating mucositis [24]. Previous studies demonstrated that flavonoids possess significant antiulcer activity [25] and also various plants containing saponins and tannins possess antiulcer activity [26]. Studies have also shown that polyphenolic compounds are beneficial in ulcers and gastrointestinal disease [27]. Although the active principle responsible for prevention of oral mucositis was not studied, it is likely that presence of flavonoid and other bioactive components may have played an important role. Toxicity studies of AESN specified no lethal effect at an oral dose of 2000 mg/kg for 14 days indicating that the extract was safe on acute administration up to 2000 mg/kg.

Chemotherapy and chemoradiotherapy cause discomfort in the mucosal lining, as a result of which difficulty in drinking, eating, and swallowing can be observed [28]. In the present study decrease in food intake and body weight was observed in both the models indicating inflammation of oral mucosa or difficulty in swallowing. However, improvement in body weight and food intake was observed in test compound treatment group, except AESN (100 mg/kg) in chemotherapy induced oral mucositis where improvement was not observed. The mucosal damage seen in high grade oral mucositis in cancer patient is represented by tongue ulceration [29]. Severity of oral mucositis is graded by oral mucositis score (OMS) and a decrease in OMS is considered as an improvement. Our results demonstrated a decrease in OMS among the treatment groups in chemoradiotherapy induced mucositis confirming a protective effect. In chemotherapy induced mucositis model standard and low dose of AESN was not very effective, whereas high dose of AESN indicated effectiveness in reducing the OMS endorsing a protective effect. In the present study, anesthesia along with radiation was employed in chemoradiotherapy induced oral mucositis, as a result of which no accidental death was observed during irradiation. However, during the 15 days of study period, 100% mortality was observed within 13th day in mucositis control group in chemoradiotherapy induced mucositis. In contrast, 0% and 17% mortality was observed in standard and treatment groups, respectively, which demonstrated the protective effect of test compound against toxicity of chemoradiotherapy. In chemotherapy induced mucositis model, 100% mortality was observed within 8th day in mucositis control group. Treatment with AESN (200 mg/kg) proved better than the lower dose in this model as the mortality rate was 0% within the study period which was at par with the standard. The risk of myelosuppression is increased when chemotherapeutic drugs like alkylating agents are given or when chemotherapy is given along with radiation therapy. Hematological parameters in our study revealed the myelosuppressive effect of chemotherapy and chemoradiotherapy. AESN did not display any restoring effect on the blood parameters especially WBC and platelets in chemoradiotherapy induced mucositis, whereas in chemotherapy induced mucositis model higher dose of AESN showed restoring effect on blood parameters, especially WBC, but no improvement in platelets was observed. Histology of tongue revealed the presence of oral mucositis, as the thickness of epithelial layer was altered and lymphocytic infiltration was observed along with reduction in number of blood vessels in chemoradiotherapy induced mucositis. Treatment with test compounds improved the thickness and higher number of blood vessels was observed indicating the protective effect of AESN in oral mucositis.

## 5. Conclusion

From the present study, we can conclude AESN at 200 mg/kg could protect oral mucositis against chemotherapy plus radiation and methotrexate induced oral mucositis. Hematological investigations could suggest that AESN (apparently) insignificantly protected chemotherapy plus radiation and methotrexate induced myelosuppression. Further investigations are required to establish whether these changes observed are caused because of altered pharmacokinetic profiles of chemotherapeutic agents or due to AESN anticlastogenic effect against these chemotherapeutic agents and radiations. Various phytochemicals including flavonoids and other bioactive components which are present in this plant are responsible for the protective effect; however, isolation of the active components was beyond the scope of present study so further studies are required to have a better insight regarding the active constituents.

## Figures and Tables

**Table 1:** Oral mucositis score (OMS) for busulfan plus radiation induced oral mucositis.

Score	Description
0	Normal
0.5	Slight pink
1.0	Slight red
2.0	Severe reddening
3.0	Focal desquamation
4.0	Exudation covering less than one half of the irradiated mucosa
5.0	Virtually complete ulceration of mucosa

**Table 2:** Oral mucositis score (OMS) for methotrexate induced oral mucositis.

Score	Description
0	Normal
1.0	Slight pink
2.0	Slight redness
3.0	Redness on tooth mucosa plus severe redness of tongue

**Table 3:** Change in hematological parameters in busulfan and radiation induced oral mucositis.

Groups	WBC (×10^3^ cells/mm^3^)	RBC (×10^6^ cells/mm^3^)	PLT (×10^3^ cells/mm^3^)
Normal control	13.66 ± 0.46	8.06 ± 0.07	612.5 ± 32.01
Mucositis control	3.8 ± 1.34^a^	6.37 ± 0.25	47.5 ± 13.95^a^
L-Glutamine	4.9 ± 1.39^a^	5.94 ± 0.42^a^	33 ± 5^a^
AESN (100 mg/kg)	3.75 ± 1.11^a^	6.38 ± 0.77	37.83 ± 4.46^a^
AESN (200 mg/kg)	6.31 ± 0.6^a^	6.74 ± 0.19	35.83 ± 3.76^a^

All values are mean ± SEM of 6 samples. ^a^ *P* < 0.05 versus normal control.

**Table 4:** Change in hematological parameter on 7th day of study period in methotrexate induced oral mucositis.

Groups	WBC (×10^3^ cells/mm^3^)	RBC (×10^6^ cells/mm^3^)	PLT (×10^3^ cells/mm^3^)
Normal control	15.02 ± 1.48	7.96 ± 0.16	583.2 ± 16.43
Mucositis control	3.0 ± 1.20^a^	7.86 ± 0.19	169.33 ± 30.02^a^
L-Glutamine	8.51 ± 0.79	6.86 ± 0.26	259.66 ± 17.79^a,b^
AESN (100 mg/kg)	8.95 ± 2.55	6.36 ± 0.61	179.66 ± 16.83^a^
AESN (200 mg/kg)	9.31 ± 1.0	6.94 ± 0.36	233.33 ± 6.65^a^

All values are expressed as mean ± SEM of 6 samples. ^a^ *P* < 0.05 versus normal control, ^b^ *P* < 0.05 versus mucositis control.

**Table 5:** Change in hematological parameter on 12th day of study period in methotrexate induced oral mucositis.

Groups	WBC (×10^3^ cells/mm^3^)	RBC (×10^6^ cells/mm^3^)	PLT (×10^3^ cells/mm^3^)
Normal control	16.1 ± 0.73	8.01 ± 0.22	583.2 ± 16.43
Mucositis control	3.0 ± 1.20^a^	7.86 ± 0.19	169.33 ± 30.02^a^
L-Glutamine	14.8 ± 1.35^b^	5.34 ± 0.28	513.21 ± 99.70
AESN (100 mg/kg)	11.12 ± 1.83^b^	5.62 ± 0.47	281.25 ± 31.83^a^
AESN (200 mg/kg)	13.6 ± 3.36^b^	5.34 ± 0.71	384.33 ± 67.17

All values are expressed as mean ± SEM of 6 samples. ^a^ *P* < 0.05 versus normal control, ^b^ *P* < 0.05 versus mucositis control.
